# Supplementary material for: Lipid regulation of hERG1 channel function
Source: Nat Commun. 2021 Mar 3;12:1409. doi: 10.1038/s41467-021-21681-8 (PMC7930123; doi:10.1038/s41467-021-21681-8)
Supplement: Supplementary file 1 — Supplementary Information [file 41467_2021_21681_MOESM1_ESM.pdf]

Supplementary information

Lipid Regulation of hERG1 Channel Function

Williams E. Miranda[1], Jiqing Guo[2], Haydee Mesa Galloso[1], Valentina Corradi[1], James P. Lees-Miller[2], D. Peter Tieleman[1*], Henry J. Duff[2*], Sergei Yu. Noskov[1*]

[1]Centre for Molecular Simulation and Department of Biological Sciences, 507 Campus Drive, University of Calgary, Alberta, Canada, T2N 4V8.

[2]Department of Cardiac Sciences, Libin Cardiovascular Institute of Alberta, 3280 Hospital Dr., University of Calgary, Alberta, Canada, T2N 4Z6

***For correspondence:** snoskov@ucalgary.ca, tieleman@ucalgary.ca, or hduff@ucalgary.ca.

Supplementary figures

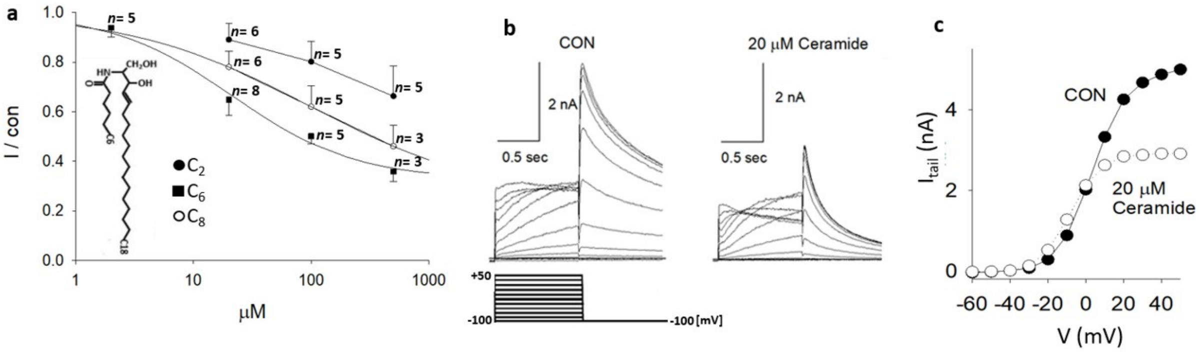

Supplementary Figure 1 Effects of ceramide application on residual currents and voltage-dependent properties of hERG1A (WT) expressed in mammalian HEK cells. a Dose-response relationship in WT at increasing concentrations of ceramides C_2, C_6 and C_8. Data are represented as mean ± SD. The number of independent experiments (n), each performed on a different cell, are indicated. The inset shows the chemical structure of a CER6 molecule. Raw data are provided in Source Data – Supplementary Fig. 1a. **b** Example of current traces before (left) and after (right) ceramide application. The voltage protocol is represented at the bottom. **c** Current-Voltage relationship of the peak tail-current. Raw data are provided in Source Data – Supplementary Fig. 1c.

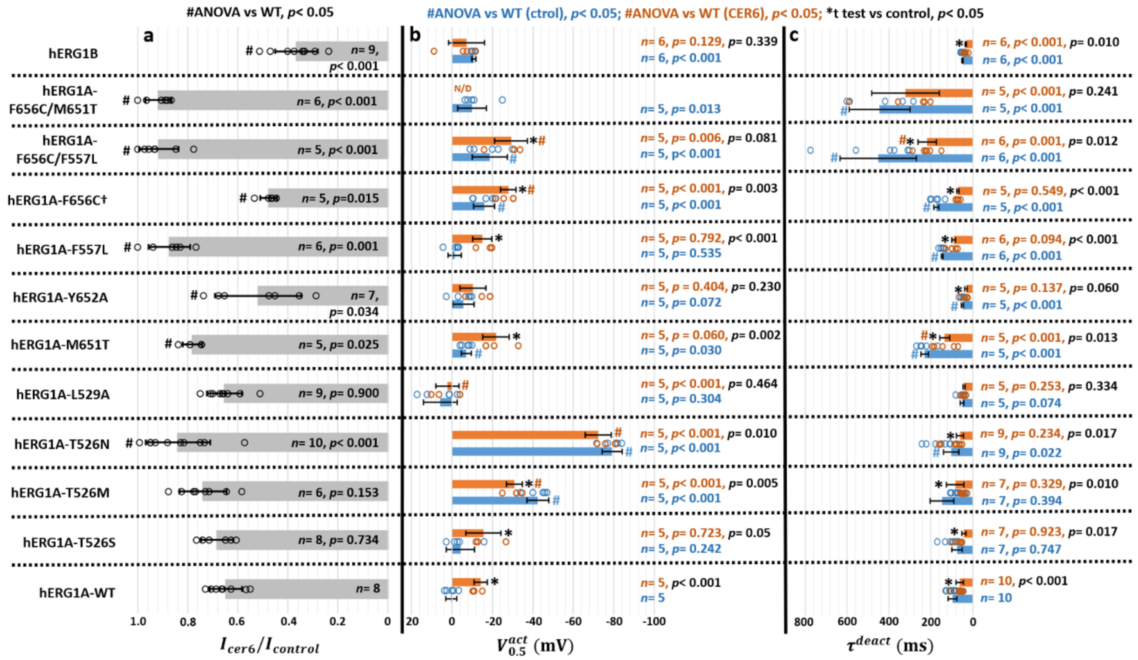

Supplementary Figure 2 Effects of hERG1 mutations on channel conduction and gating properties. a Residual currents after CER6 application. Panels **b** and **c** display half-activation voltages and deactivation τ values, respectively (blue: control, orange: after ceramide application). N/D: not determined. Data are represented as mean ± SD in **a-c**. The number of independent experiments (n) in **a-c**, each performed on a different cell, are indicated. The *p* values evaluated by one-way ANOVA with tukey test for each hERG1-variant vs WT are shown in **a-c**, where the symbol '#' indicates $p< 0.05$. The two-tailed *p* values evaluated by paired t-test (CER6 vs control) for each hERG1-variant are shown in **b-c**, where the symbol '*' indicates $p< 0.05$ (black color). Ceramide concentration of 20 μM was used for all hERG1-variants, except for F656C (†400 μM). A voltage of -100 mV was used for measuring peak-tail currents for all channel-variants, except for hERG1B (-50 mV). Raw data, confidence intervals, t-values and degrees of freedom are provided in Source Data – Supplementary Fig. 2a-c.

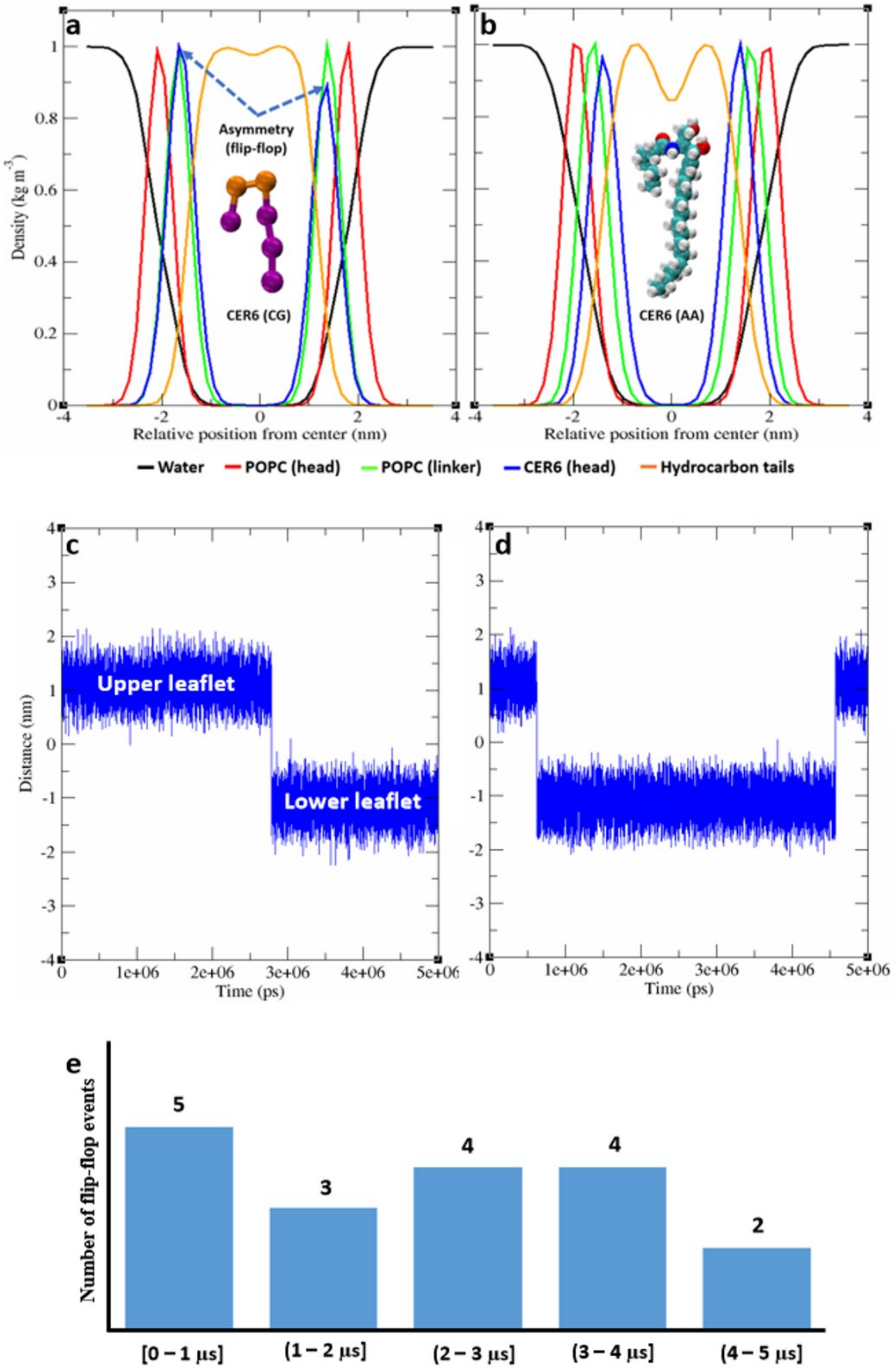

Supplementary Figure 3 Distribution of lipids between the upper and lower leaflets in protein-free bicomponent bilayers (POPC:CER6 = 8:2). Panels **a** and **b** show the density profiles (1D) along the membrane normal for CG and AA systems, respectively. The densities for specific chemical groups analyzed are shown in different colors (see legend at the bottom of the figure). Structural models of CER6 are shown at the center of each panel. Ceramide CG beads (**a**) are colored according to their polarity (orange: polar, purple: apolar). Ceramide C, N, H, and O atoms (**b**) are colored cyan, blue, white, and red, respectively. Raw data for these graphs are provided in Source Data – Supplementary Fig. 3a, b. Panels **c** and **d** show examples of flip-flop events for ceramides in the CG-MD simulation. A histogram for the number of flip-flop events per microsecond of CG-MD trajectory is shown in panel **e**.

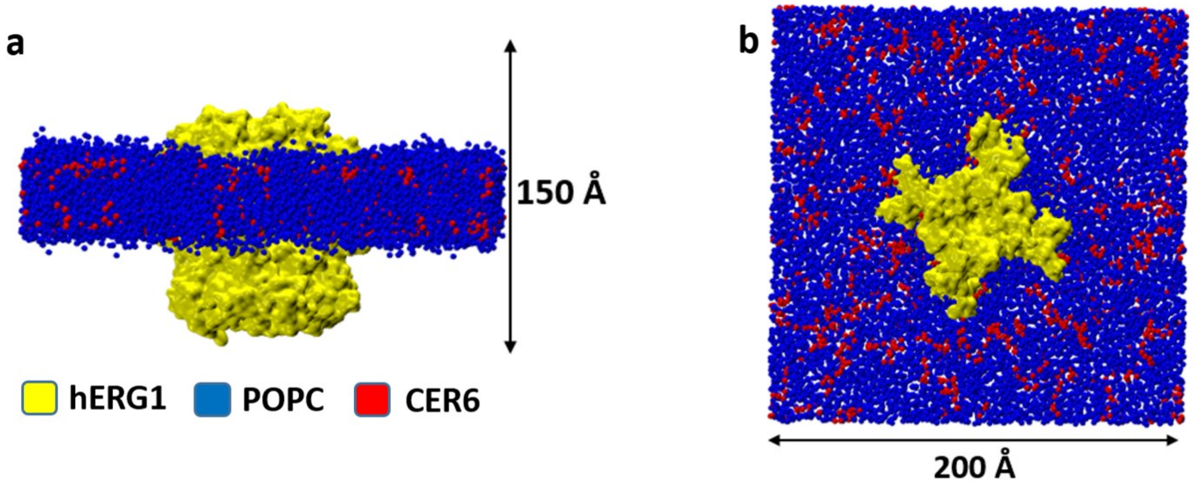

Supplementary Figure 4 Representation of coarse-grained system for WT open-state (PDBID: 5VA2). The panels **a** and **b** show side- and upper- views of the system, respectively, where the channel (yellow surface) is embedded in a membrane patch composed of POPC and ceramide (blue and red beads, respectively). The dimensions of the system are indicated by double-headed arrows.

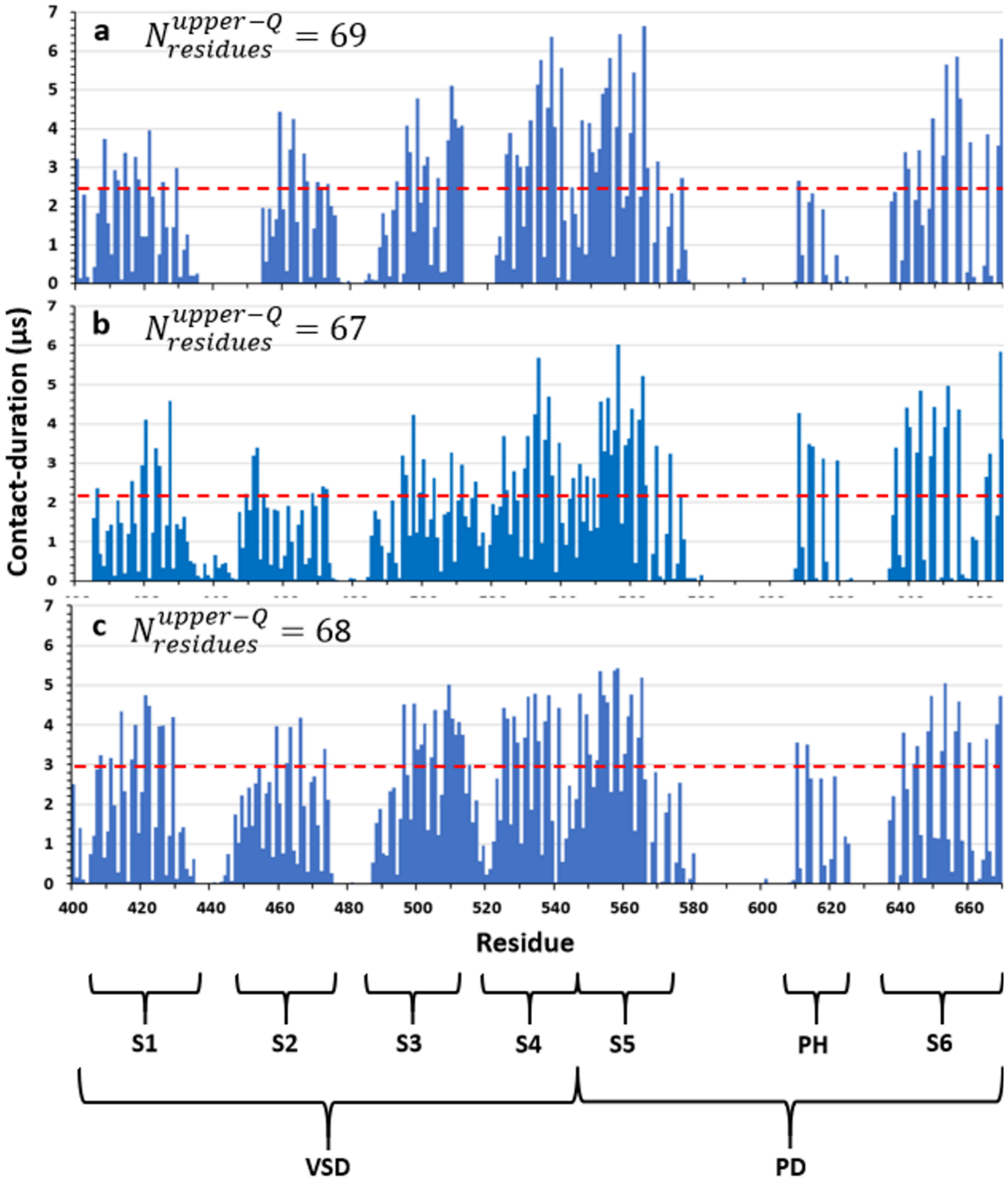

Supplementary Figure 5 Average contact-duration per residue for hERG1A-variants in CG-MD simulations. a WT open-state. **b** WT closed PD state. **c** F656C/F557 open-state. In each panel, the red line indicates the lower limit of the upper quartile. The number of residues within the upper quartile is shown at the top of each panel. The achronyms S1-S6 indicate the location of transmembrane helices, PH indicates pore-helix. The voltage-sensing and pore domains are indicated as VSD and PD, respectively. Per-residue contact duration values and quartile analysis are provided in Source Data – Supplementary Fig. 5.

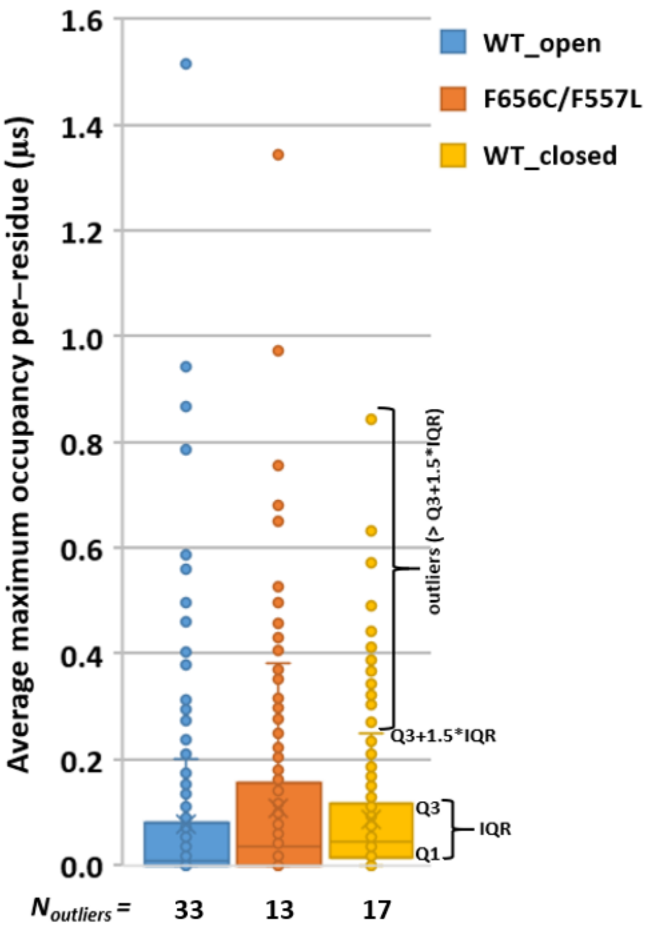

Supplementary Figure 6 Average maximum occupancy per-residue from CG-MD simulations for the hERG1A-variants. WT open-state (blue), F656C/F557L open-state (orange) and WT closed PD state (yellow). Data are represented as boxplots where the middle line is the median, the lower and upper hinges correspond to the first and third quartiles, the upper whisker extends from the hinge to the largest value no further than 1.5 × IQR from the hinge (where IQR is the interquartile range) and the lower whisker extends from the hinge to the smallest value at most 1.5 × IQR of the hinge, while data beyond the end of the whiskers are outlying points that are plotted individually. The number of maximum-occupancy outliers per system is indicated below. An example of outliers' identification is depicted for the yellow boxplot. Maximum occupancy values per-residue and quartile analysis are provided in Source Data – Supplementary Fig. 6.

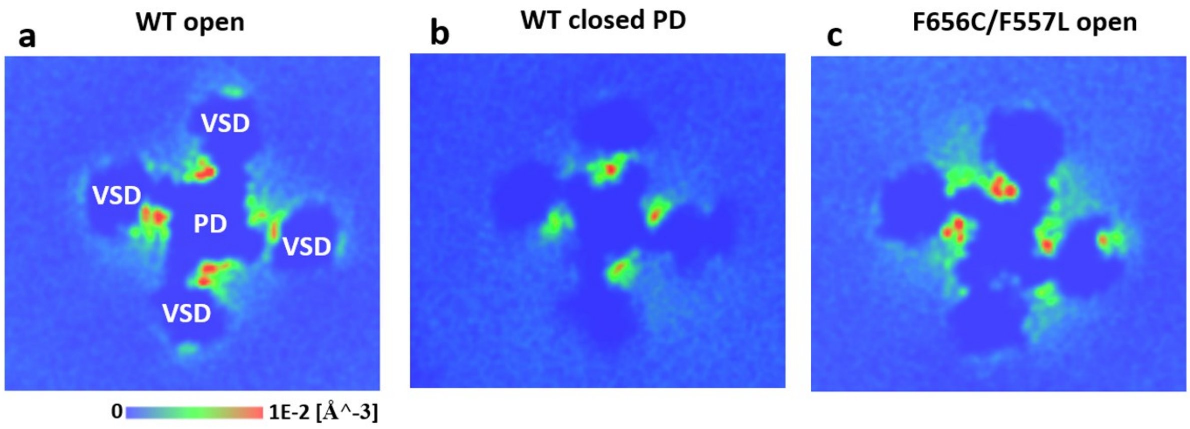

Supplementary Figure 7 Average ceramide density maps (2D), normal to the membrane plane. a WT open-state. **b** WT closed PD state. **c** F656C/F557L open-state. Low, medium, and high-density regions are highlighted in blue, green, and red, respectively. The VSDs and PD indicate the voltage-sensing and pore domains, respectively. Each slice was taken using the same z coordinate at the level of residues 557, 651, and 656, which are spatially close in the structure (see Fig. 1a-inset).

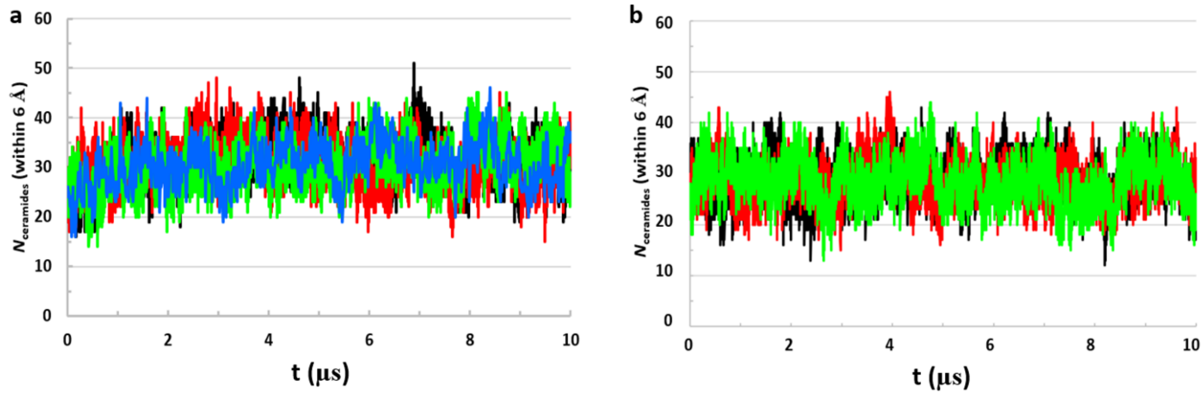

Supplementary Figure 8 Total ceramides in contact with the channel (within 6 Å) along CG-MD trajectories. a WT open state. **b** WT closed PD state. For each system, the replicas are represented by a different color. The raw data for both graphs are provided in Source Data – Supplementary Fig. 8.

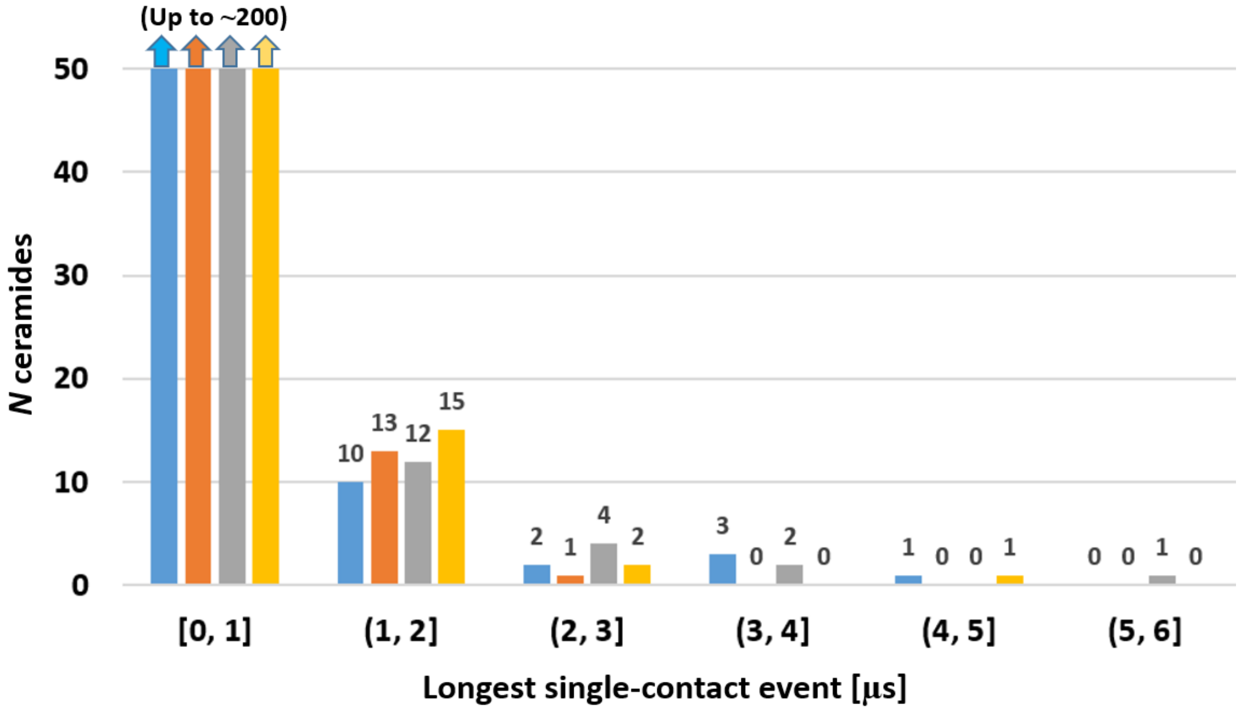

Supplementary Figure 9 Histogram of the longest single-contact event for each ceramide molecule from CG-MD simulations of WT open-state. Each color represents a different replica. The raw data for each replica are provided in Source Data – Supplementary Fig. 9.

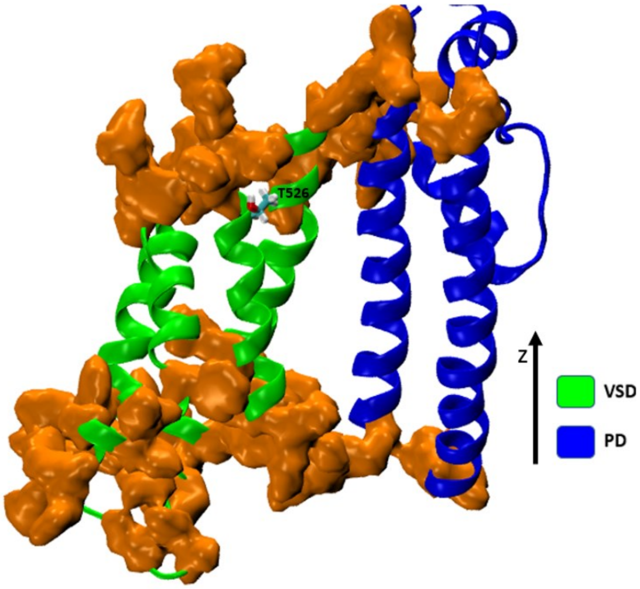

Supplementary Figure 10 Contact map for phosphatidylcholine headgroups in CG-MD simulations of hERG1-WT (open state). Residues identified as outliers from the maximum-occupancy analysis are highlighted as orange surface. The sidechain of residue T526 (only contacted by ceramides headgroup) is shown in licorice representation, where the C, O, and H atoms are colored in cyan, red, and white, respectively. The black arrow indicates the axis in Z. The VSD and PD of the channel (one monomer, side view) are highlighted in green and blue cartoons, respectively. The maximum-occupancy values per-residue for popc and cer6 headgroups are provided in Source Data – Supplementary Fig. 10.

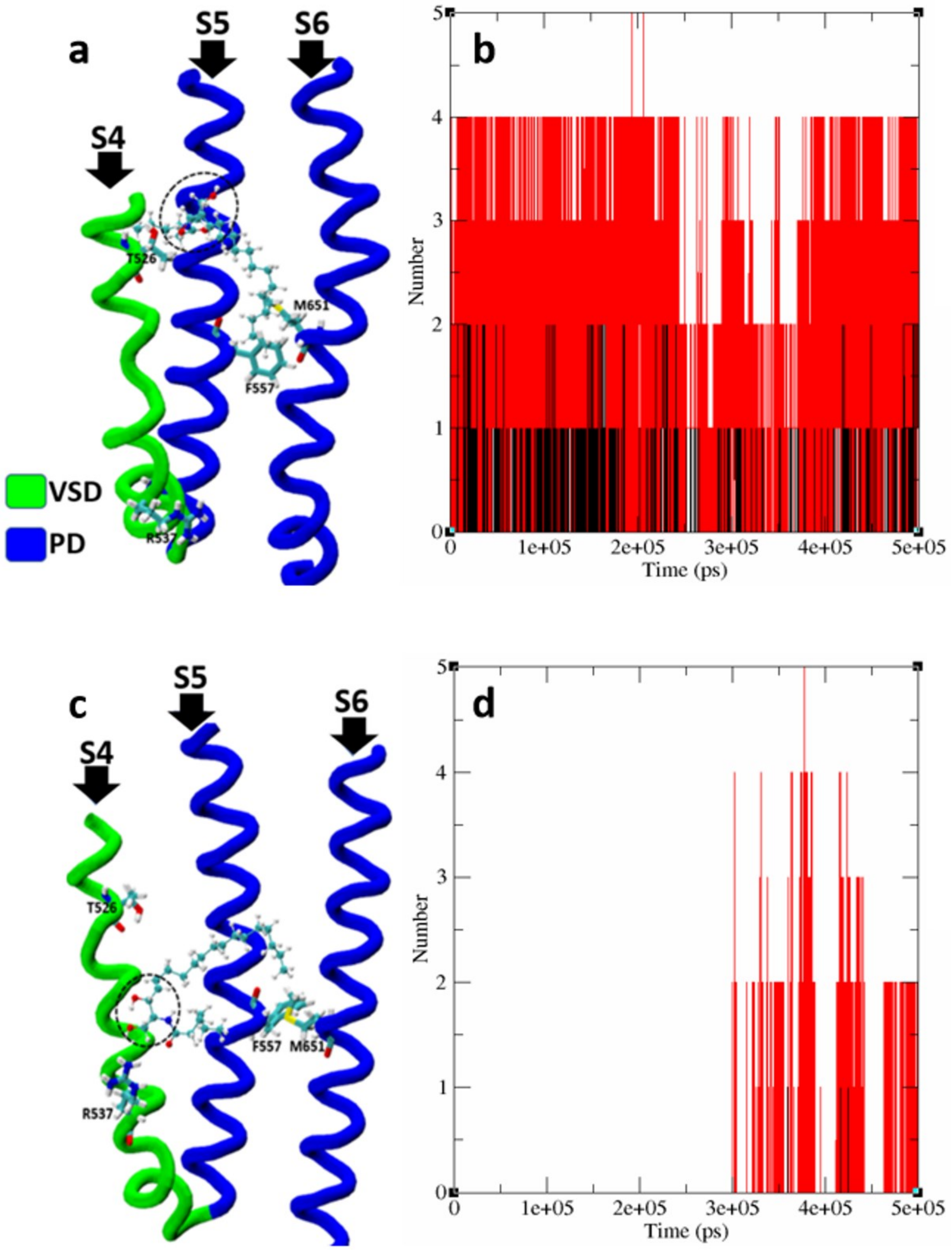

Supplementary Figure 11 Hydrogen-bonds established by ceramide headgroup at the VSD-PD interface of WT open-state in AA-MD simulations. Panels **a** and **c** show the helices S4-S6 in tube representation. Those corresponding to the VSD and the PD are highlighted in green and blue, respectively. A ceramide molecule is shown in balls and sticks representation and its polar headgroup is enclosed within a dashed black circle. Residues T526, R537, M651, and F557 are shown in licorice representation. The C, H, N, O, and S atoms from these residues and the ceramide molecule are highlighted in cyan, white, blue, red, and yellow, respectively. Panels **b** and **d** show the dynamics of the most stable interactions found among the five replicas (500 ns each) for T526-ceramide and R537-ceramide, respectively. The red lines in both panels indicate the number of hydrogen bond pairs within 3.5 Å between the headgroup from a single ceramide molecule and the residue T526 from one of the VSDs. According to the graph, this number has a maximum value of five. The raw data for both graphs are provided in Source Data – Supplementary Fig. 11.

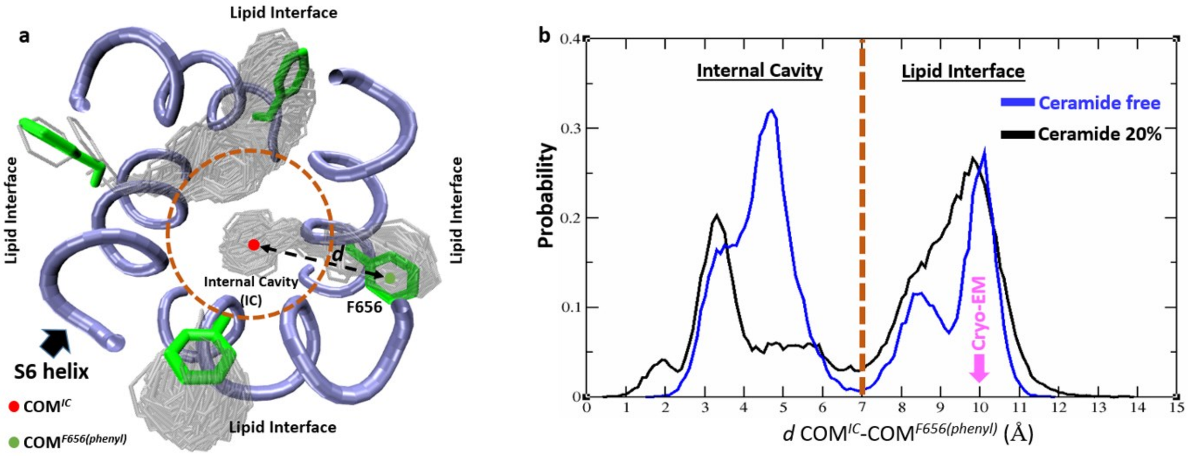

Supplementary Figure 12 Dynamics of F656 sidechains in WT open-state AA-MD simulations. a The sidechains are shown in licorice representation, highlighting the initial state (solid green) and snapshots (transparent silver) from one replica (500 ns long). The orange dashed-circle delimits the internal cavity of the channel. The S6 helices containing F656 residues are shown in tube representation, iceblue color. The red and green dots indicate the center of mass (COM) of the internal cavity (IC) and the COM of the phenyl moiety from one of the four F656 sidechains in the Cryo-EM structure, respectively. The dashed double-headed arrow indicates the distance (*d*) between both COMs. **b** Combined distribution for *d* among all four F656 sidechains for hERG1 inserted in pure POPC bilayer (blue color, 4 replicas x 2 microseconds each) and binary POPC-CER6 mixture (black color, 20 replicas x 500 nanoseconds each). The arrow (magenta) indicates the reference *d* value from the Cryo-EM structure (PDBID: 5VA2). The raw data for the distributions shown in panel B are provided in Source Data – Supplementary Fig. 12.

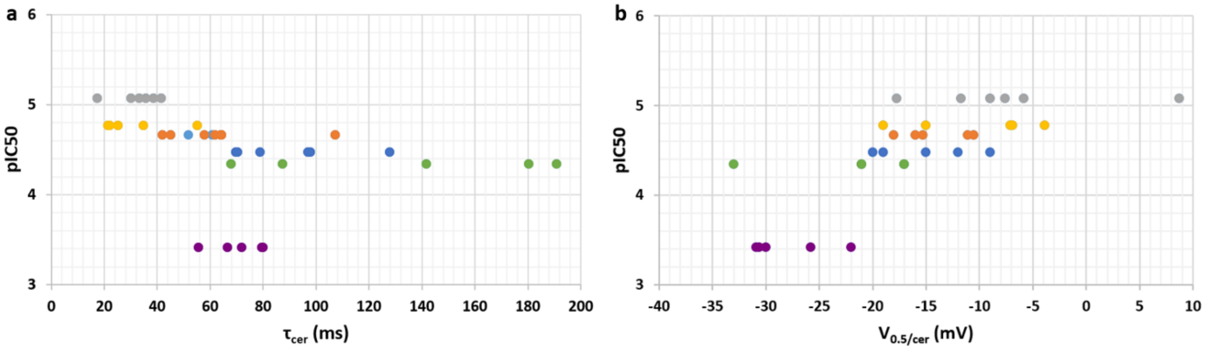

	hERG1A WT	hERG1B	hERG1A Y652A	hERG1A M651T	hERG1A F557L	hERG1A F656C
τ	n= 10	n= 6	n= 5	n= 5	n= 6	n= 5
$V_{0.5}$	n= 5	n= 6	n= 5	n= 5	n= 5	n= 5

Supplementary Figure 13 Gating properties of hERG1 variants -expressed in mammalian HEK cells- after ceramide application. Scatter plots for pIC50 (-log[IC50]) vs the change in deactivation τ (**a**) and activation $V_{0.5}$ (**b**) after ceramide application (see Source Data – Supplementary Fig. 2b, c). Deactivation τ values in **a** were measured at -100 mV, except for hERG1B (-50 mV). [CER6] = 20 µM was used for all measurements shown in panels **a** and **b**, except for the mutant hERG1A-F656C (400 µM). The legend for scatter plots in **a** and **b** are shown at the bottom: hERG1A- WT (orange), Y652A (yellow), M651T (green), F557L (blue), F656C (purple) and hERG1B (gray). The table at the bottom indicates the number of independent experiments (n), each performed on a different cell.

**Supplementary Table 1** Primers used for site-directed mutagenesis.	
**Substitution**	**Primer sequence**
T526M	*FORWARD:* GCTGAAGATGGCGCGGCTGCT<br>*REVERSE:* GCCGCGCCATCTTCAGCAG
T526S	*FORWARD:* TCGGGCTGCTGAAGTCTGCGCGGCT<br>*REVERSE:* CTTCAGCAGCCCGATC
T526N	*FORWARD:* TCGGGCTGCTGAAGAATGCGCGGCT<br>*REVERSE:* CTTCAGCAGCCCGATC
L529A	*FORWARD:* CTGCGCGGGCGCTGCGGCT<br>*REVERSE:* GCCGCAGCGCCCGCGCAGT
F557L	*FORWARD:* GTGCACCCTTGCGCTCA<br>*REVERSE:* GAGCGCAAGGGTGCACA
M651T	*FORWARD:* GCTCCCTCACGTATGCTAGCA<br>*REVERSE:* CTAGCATACGTGAGGGAGCCA
Y652A	*FORWARD:* CCCTCATGGCTGCTAGCATC<br>*REVERSE:* GCTAGCAGCCATGAGGGA
F656C	*FORWARD:* CTAGCATCTGCGGCAACGT<br>*REVERSE:* CGTTGCCGCAGATGCTAGCA

Note: The double mutants were made using the same primers as the corresponding single mutants.
