# Supplementary material for: Lipid regulation of hERG1 channel function
Source: Nat Commun. 2021 Mar 3;12:1409. doi: 10.1038/s41467-021-21681-8 (PMC7930123; doi:10.1038/s41467-021-21681-8)
Supplement: Supplementary file 3 — Reporting Summary [file 41467_2021_21681_MOESM3_ESM.pdf]

nature research

Corresponding author(s): Sergei Noskov, Henry Duff and Peter Tieleman

Last updated by author(s): Jan 16, 2021

Reporting Summary

Nature Research wishes to improve the reproducibility of the work that we publish. This form provides structure for consistency and transparency in reporting. For further information on Nature Research policies, see our Editorial Policies and the Editorial Policy Checklist.

Statistics

For all statistical analyses, confirm that the following items are present in the figure legend, table legend, main text, or Methods section.

n/a	Confirmed	
☐	☒	The exact sample size (n) for each experimental group/condition, given as a discrete number and unit of measurement
☐	☒	A statement on whether measurements were taken from distinct samples or whether the same sample was measured repeatedly
☐	☒	The statistical test(s) used AND whether they are one- or two-sided<br>*Only common tests should be described solely by name; describe more complex techniques in the Methods section.*
☐	☒	A description of all covariates tested
☐	☒	A description of any assumptions or corrections, such as tests of normality and adjustment for multiple comparisons
☐	☒	A full description of the statistical parameters including central tendency (e.g. means) or other basic estimates (e.g. regression coefficient) AND variation (e.g. standard deviation) or associated estimates of uncertainty (e.g. confidence intervals)
☐	☒	For null hypothesis testing, the test statistic (e.g. F, t, r) with confidence intervals, effect sizes, degrees of freedom and P value noted<br>*Give P values as exact values whenever suitable.*
☒	☐	For Bayesian analysis, information on the choice of priors and Markov chain Monte Carlo settings
☒	☐	For hierarchical and complex designs, identification of the appropriate level for tests and full reporting of outcomes
☒	☐	Estimates of effect sizes (e.g. Cohen's d, Pearson's r), indicating how they were calculated

Our web collection on statistics for biologists contains articles on many of the points above.

Software and code

Policy information about availability of computer code

Data collection	GROMACS (v5.0.7) to perform CG-MD and AA-MD simulations and free energy calculations. Clampex (v9.2) to process data signal in e-physiology. CHARMM-GUI for CG systems' setup.
Data analysis	GROMACS (v5.0.7) built-in tools for trajectory analysis; Grid-MAT (v2.0) for surface area calculation (per lipid type); VMD (v1.9.3) for visualizations and clustering analysis; For experimental data analysis (electrophysiology) the following software was used: Clampfit (v8.2 and v9.2) and Excel 2016. For experimental data display: Sigmaplot (v14.0).

For manuscripts utilizing custom algorithms or software that are central to the research but not yet described in published literature, software must be made available to editors and reviewers. We strongly encourage code deposition in a community repository (e.g. GitHub). See the Nature Research guidelines for submitting code & software for further information.

Data

Policy information about availability of data

All manuscripts must include a data availability statement. This statement should provide the following information, where applicable:

- Accession codes, unique identifiers, or web links for publicly available datasets
- A list of figures that have associated raw data
- A description of any restrictions on data availability

Source data for each figure are provided with this paper. The simulation trajectories will be made available upon requests.

Field-specific reporting

Please select the one below that is the best fit for your research. If you are not sure, read the appropriate sections before making your selection.

[x] Life sciences   [ ] Behavioural & social sciences   [ ] Ecological, evolutionary & environmental sciences

For a reference copy of the document with all sections, see nature.com/documents/nr-reporting-summary-flat.pdf

Life sciences study design

All studies must disclose on these points even when the disclosure is negative.

Sample size	All of the comparisons were prespecified and all of the comparisons and all of the data are reported. In order to prospectively calculate sample size, an estimate of the expected mean change is necessary. However, the present study was exploratory, thus there was no a priori reason to consider the impact of specific mutations on IC50. Importantly, previous studies had not ascertained complete IC50 values; instead most studies examined the effects of fixed concentrations of ceramide. In addition, the concentrations of ceramide that are testable are limited by solubility in the Tyrodes solution. There was no prospective way of predicting the IC50 concentrations using patch clamp studies except by exploratory methods and also limited by the solubility of ceramide. In addition, we acknowledge that there may be other mutations, unknown to us at this time, which could be relevant. A priori, we generally required a minimum of n= 5 independent experiments for each point on each IC50 curve. However, the n values near the IC50 point and near the maximum blockade have a major impact on the reliability of the IC50 estimate. Frequently we increased the n values at these putative points to be more certain about the reliability of the measurement. The n values for each point of the concentration-response relationship are presented in the figure legends.
Data exclusions	No data were excluded from the analysis.
Replication	All results were included in the analysis and are reported. The only reasons that data were excluded from analysis were: 1) loss or an unstable giga-seal or access resistance change during baseline or drug -superfusion or 2) if there was progressive run-down of the current at baseline.<br><br>The n values of each experiments show the times of each experiments replicated independently. All data of replication are included in the analysis. The only reason data are not analyzed and not reported are described above. Since little or measurable block at [CER6]= 100 uM was observed for F557L/F656C, the block value at [CER6] = 2 uM was not examined.
Randomization	This study was exploratory in design. Experiments on the single mutations preceded those of the double the mutations. The sequence of examination of the single mutations was random except based on the availability of the transfection constructs. The availability of constructs was defined by our molecular biology source library of mutations. If the construct needed to be resynthesized and thus was not available, the next single mutation was examined and when the construct was available, the impact of that single mutation examination was examined. The construct availability and thus feasibility could limit the choice of the mutation to be examined each week.
Blinding	Our patch clamp team currently consists of a single experimentalist (JG). This experimentalist is responsible for transfection of the cells and subsequent patch clamp studies. Given the fact that our experimental laboratory consists of a single person, blinding is not feasible.

Reporting for specific materials, systems and methods

We require information from authors about some types of materials, experimental systems and methods used in many studies. Here, indicate whether each material, system or method listed is relevant to your study. If you are not sure if a list item applies to your research, read the appropriate section before selecting a response.

Materials & experimental systems

n/a	Involved in the study	
[x]	[ ]	Antibodies
[ ]	[x]	Eukaryotic cell lines
[x]	[ ]	Palaeontology and archaeology
[x]	[ ]	Animals and other organisms
[x]	[ ]	Human research participants
[x]	[ ]	Clinical data
[x]	[ ]	Dual use research of concern

Methods

n/a	Involved in the study	
[x]	[ ]	ChIP-seq
[x]	[ ]	Flow cytometry
[x]	[ ]	MRI-based neuroimaging

Eukaryotic cell lines

Policy information about cell lines

Cell line source(s)	HEK293 cell-line was originated from Ron Kopito's lab at Stanford University. hERG1 expressing human myocyte cell-line was from Dr. Houghton's lab at the University of Alberta. We obtained this cell-line from the Lytton laboratory as previously reported - M Tsoi, K H Rhee, D Bungard, X F Li, S L Lee, R N Auer, J Lytton Molecular cloning of a novel potassium-dependent sodium-calcium exchanger from rat brain J Biol Chem. 1998 Feb 13;273(7):4155-62.
Authentication	The cell line that was used was a commercial HEK-293 cell line used stably in the lab for the last 5 years. No further characterization of the cell lines were performed. hERG1- myocyte cell line was used in the lab for last 2 years and we have performed electrophysiology screening experiments with this cell line regularly. The myocyte cell line allows for stable hERG1 signal and show almost no other endogenous currents.
Mycoplasma contamination	HEK293 cell line was tested negative for mycoplasma contamination by Dr. Jonathan Lytton's lab. The hERG1 expressing human myocyte cell-line was tested negative for mycoplasma contamination by Houghton's lab.
Commonly misidentified lines (See ICLAC register)	Only HEK cells (with a special note that this cell line is different from HEK-293) are listed in latest version 10.0 of ICLAC register as potentially misidentified lines. The ICLAC made a specific note that HEK-293 cell lines are not impacted by HeLa cell line contamination. Therefore, no specific effort was made to check HEK-293 commercial line used in our study for potential misidentifications or contamination. The hERG1 myocyte cell line is not listed in the latest version of 10.0 ICLAC. We tested this cell line with standard set of CiPA compounds and found excellent agreement with data published with other cell lines.
